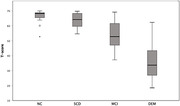# Cross‐cultural adaptation and validation of everyday functioning for traditional Chinese in Taiwan

**DOI:** 10.1002/alz.090707

**Published:** 2025-01-09

**Authors:** Poyu Chen, Chin‐Yi Wu, I‐Ching Chuang, Yu‐hua Huang, Jung‐Lung Hsu, Sietske A.M Sikkes

**Affiliations:** ^1^ Chang Gung University, Taipei Taiwan; ^2^ Chang Gung University, Taoyuan, NA Taiwan; ^3^ Chang Gung Memorial Hospital, Taoyuan, NA Taiwan; ^4^ TuCheng Hospital, New Taipei City Taiwan; ^5^ Alzheimer Center Amsterdam, Neurology, Vrije Universiteit Amsterdam, Amsterdam UMC, Amsterdam Netherlands

## Abstract

**Background:**

Detecting functional performance decline from normal aging to dementia is critical for early detection and intervention. The informant‐based A‐IADL‐Q‐SV has been demonstrated with good psychometric properties. The aims of this study were to: (1) translate, culturally adapt, and validate the Traditional Chinese version of the A‐IADL‐Q‐SV‐TC for Taiwanese; (2) evaluate the psychometric properties.

**Methods:**

We conducted the forward and backward translations with culture adaptation, followed by the consensus meeting with the original developer. The psychometric properties were investigated in both community and memory clinic settings. The Mini‐Mental State Examination (MMSE) and ICF‐based IADL scales were evaluated to test the construct validity and concurrent validity, respectively. Furthermore, diagnoses were established in a multidisciplinary consensus meeting, independent of the A‐IADL‐Q‐SV‐TC scores. The discriminate validity was examined by investigating the difference in A‐IADL‐Q‐SV‐TC between diagnostic groups across the dementia spectrum as well as the optimal cut‐off score to differentiate MCI from cognitively intact populations.

**Results:**

146 informants of participants with normal cognition (NC), subjective cognitive decline (SCD), mild cognitive impairment (MCI), and dementia participated this study. Results showed strong correlation between A‐IADL‐Q‐SV‐TC T‐score and both MMSE (r = .850, p <.001) and ICF‐based IADL scales (CDI, r = ‐.874, p <.001; DI, r = ‐.903, p <.001). As for the discriminate validity, participants diagnosed with cognitive impairment had lower A‐IADL‐SQ T‐scores (M= 45. 68, SD = 13.92) than participants without cognitive impairment (M = 65.03, SD = 4.53), t (85.521) = 11.227, p < .001. Furthermore, the overall diagnostic accuracy based on the AUC was 0.915 (95% CI: 0.87‐ 0.96) for the A‐IADL‐Q‐SV‐TC. The optimal cut‐off score for A‐IADL‐Q‐SV‐TC to differentiate MCI from cognitively intact populations was 54.58, resulting in a sensitivity of 0.72 and specificity of 0.98.

**Conclusions:**

We demonstrated the A‐IADL‐SQ‐TC has sound psychometric properties as a valuable diagnostic tool.